# Preference for C_4_ shade grasses increases hatchling performance in the butterfly, *Bicyclus safitza*

**DOI:** 10.1002/ece3.2235

**Published:** 2016-06-29

**Authors:** Ossi Nokelainen, Brad S. Ripley, Erik van Bergen, Colin P. Osborne, Paul M. Brakefield

**Affiliations:** ^1^ Department of ZoologyUniversity of CambridgeCambridgeCB2 3EJUK; ^2^ Department of BotanyRhodes UniversityP.O. Box 94Grahamstown6140South Africa; ^3^ Department of Animal and Plant SciencesUniversity of SheffieldSheffieldS10 2TNUK

**Keywords:** C_3_ grass, C_4_ grass, female preference, host selection, offspring performance, optimal oviposition theory, plant–herbivore interaction, preference–performance hypothesis

## Abstract

The Miocene radiation of C_4_ grasses under high‐temperature and low ambient CO_2_ levels occurred alongside the transformation of a largely forested landscape into savanna. This inevitably changed the host plant regime of herbivores, and the simultaneous diversification of many consumer lineages, including *Bicyclus* butterflies in Africa, suggests that the radiations of grasses and grazers may be evolutionary linked. We examined mechanisms for this plant–herbivore interaction with the grass‐feeding *Bicyclus safitza* in South Africa. In a controlled environment, we tested oviposition preference and hatchling performance on local grasses with C_3_ or C_4_ photosynthetic pathways that grow either in open or shaded habitats. We predicted preference for C_3_ plants due to a hypothesized lower processing cost and higher palatability to herbivores. In contrast, we found that females preferred C_4_ shade grasses rather than either C_4_ grasses from open habitats or C_3_ grasses. The oviposition preference broadly followed hatchling performance, although hatchling survival was equally good on C_4_ or C_3_ shade grasses. This finding was explained by leaf toughness; shade grasses were softer than grasses from open habitats. Field monitoring revealed a preference of adults for shaded habitats, and stable isotope analysis of field‐sampled individuals confirmed their preference for C_4_ grasses as host plants. Our findings suggest that plant–herbivore interactions can influence the direction of selection in a grass‐feeding butterfly. Based on this work, we postulate future research to test whether these interactions more generally contribute to radiations in herbivorous insects via expansions into new, unexploited ecological niches.

## Introduction

During the Miocene, the spread and diversification of grasslands dominated by plants using the C_4_ carbon‐fixing photosynthetic pathway generated the African savanna biome (Cerling et al. 1997; Osborne and Beerling 2006; Edwards et al. 2010; Spriggs et al. 2014). While the advantages of C_4_ plants in these habitats over species using the ancestral C_3_ photosynthetic pathway are understood in terms of physiological adaptations to high‐temperature and low ambient CO_2_ levels (Laetsch 1974; Ehleringer and Monson 1993; Sage 2004; Ripley et al. 2007; Taylor et al. 2010, 2014; Christin and Osborne 2014), the consequences of the biome transition for many grazer communities remain less well known (Caswell et al. 1973; Caswell and Reed 1976; Boutton et al. 1978; Fagerstone and Williams 1982; Barbehenn et al. 2004a; van Bergen et al. 2016). However, the co‐occurring radiation of the grass‐feeding *Bicyclus* butterflies together with the fragmentation of forests, spread of savanna, and evolution of C_4_ grasses (Peña and Wahlberg 2008) suggests that these two radiations may be linked; one potential mechanism being the associated plant–herbivore interactions. Here, using *Bicyclus safitza* as a widespread representative species of the genus, we seek to understand how dietary and oviposition preference for sun or shade grasses with either C_3_ or C_4_ photosynthetic physiology has consequences for butterfly fitness.

The spread of the savanna biome divided sub‐Saharan habitats broadly into C_3_‐dominated forests and C_4_‐dominated savannas (Osborne and Beerling 2006; Beerling and Osborne 2006; Edwards et al. 2010). This may then have caused diverging trajectories between butterflies and their host plants. *Bicyclus* butterflies are normally found in closed, shaded woodland habitats, which are typically dominated by C_3_ plants, but some species can be found in open, sunlit C_4_‐dominated grassy habitats (Brakefield and Reitsma 1991; Roskam and Brakefield 1999). From the perspective of egg‐laying butterflies, adult females need to efficiently find the correct habitat patch to locate suitable host plants for oviposition and larval development, because searching for host plants may be costly in time and energy (Thompson 1988; Thompson and Pellmyr 1991; Gripenberg et al. 2010; Knolhoff and Heckel 2014; Schäpers et al. 2015). Hatching larvae on the other hand are dependent on the choice of the female parent, as they have limited mobility and require a suitable host plant for growth and survival (Thompson and Pellmyr 1991; Knolhoff and Heckel 2014). Maternal oviposition choice is thus predicted to maximize offspring fitness, because selection has shaped innate female preferences (i.e., the preference–performance hypothesis, Jaenike 1978; Valladares and Lawton 1991; West and Cunningham 2002).

The different physiological adaptations of host plants may result in contrasting utilization costs for herbivores (Caswell et al. 1973; Caswell and Reed 1976). C_4_ leaves tend to be tougher than C_3_ leaves due to a higher density of leaf veins, fiber bundles, and silica phytoliths (e.g., Laetsch 1974; Caswell and Reed 1976; Boutton et al. 1978; Barbehenn and Bernays 1992; Massey and Hartley 2006; Massey et al. 2007). In addition, C_4_ leaves have lower nutritional values than C_3_ leaves because of lower nitrogen and higher carbon content (Barbehenn et al. 2004a,b). Therefore, the harvesting and processing costs of feeding on C_4_ leaves should be significantly higher, and the digestibility and nutritional value lower than a diet of C_3_ leaves. On the other hand, it is possible that plant adaptations to the respective abiotic environment (e.g., sun vs. shade) may contribute to its palatability to herbivores. For example, narrow, tough leaves and the high fiber content of sun‐adapted plant leaves may prevent consumption by herbivores, whereas the larger, softer leaves of shade‐adapted plants may make them more attractive to herbivores. In spite of this, selective herbivores can be expected to avoid a C_4_ plant diet when C_3_ plants are available, and will develop more slowly and have lower fitness on a diet of C_4_ than on C_3_ leaves (Caswell et al. 1973; Heidorn and Joern 1984; Barbehenn et al. 2004a; Christin and Osborne 2014). These predictions have not always received support from experimental data (e.g., Fagerstone and Williams 1982; Barbehenn and Bernays 1992; van Bergen et al. 2016), which suggests that subtleties in host–plant interactions can be complex.

Here, we examine host plant ecology of the Common Bush Brown (*Bicyclus safitza*) butterfly in South Africa, which is the most southerly part of the range of this species. First, we examine whether ovipositing females prefer local species of C_3_ or C_4_ grasses that grow in open or shaded habitats. Secondly, we compare hatchling establishment on these grass species in terms of growth and survival, and measure the leaf characteristics of host plants in terms of accessibility and palatability. Thirdly, we study in the field the occurrence of adults in different habitats and their isotope signatures which provide direct evidence of C_3_ and C_4_ plants in the larval diet due to detectable differences in the relative amount of carbon (*δ* ^13^C) isotopes (Boutton et al. 1978; Ehleringer and Monson 1993; Cerling et al. 1997). As *B. safitza* can be found both in forests and more open grassland habitats, we predict that in forests where C_3_ grasses dominate the understory the individuals should be C_3_ specialists, whereas in drier, more open environments dominated by C_4_ grasses, individuals should be more opportunistic in their host plant choice.

## Materials and Methods

### Study animals & plants

The study was conducted from October 2014 until April 2015 in the Eastern Cape of South Africa. The laboratory stock of *B. safitza* originated from 55 wild‐caught butterflies collected in mid‐November from Kasouga (−33.650250°, 26.740267°, coordinates in decimal degrees; permit numbers CRO12/14CR, CRO13/14CR, and RA‐0198). Butterflies were taken into the greenhouse at the nearby Department of Botany, Rhodes University, Grahamstown. Conditions in the greenhouse were maintained at approximately 27°C and 65% relative humidity (RH), with a natural light–dark cycle. Butterflies were kept in large insect rearing cages (Insectopia, Austrey, UK) and provided with fermenting banana ad libitum. Individuals mated freely and were provided with *Ehrharta erecta* (C_3_) and *Brachiaria chusquoides* (C_4_) grasses for oviposition. Eggs were collected and larvae reared on wheat from hatching (*Triticum aestivum*, C_3_).

A stock of wild grasses was collected from the surroundings of Grahamstown, potted, and maintained outdoors under shade cloth. The plants were allowed to habituate for approximately a week in pots, before entering the experiment. We chose 12 common, local species to represent ecologically relevant host plants, all of which could potentially be encountered by adult butterflies in the wild; three each from either open (O) or shade (S) environments, and with either C_3_ or C_4_ photosynthetic physiology. The species were as follows. C_3_O: *Allopteropsis semialata ssp. eckloniana, Panicum ecklonii, Merxmuellera disticha*. C_3_S: *Ehrharta erecta, Oplismenus hirtellus, Panicum aequinerve*. C_4_O: *Allopteropsis semialata ssp. semialata, Hyparrhenia hirta, Brahiaria serrata*. C_4_S: *Brachiaria chusquoides, Dactyloctenium australe, Panicum deustum*. The identity of plants and their photosynthetic pathway were confirmed from herbarium samples at the Schönland Herbarium, Rhodes University, and by stable isotope analysis of dried leaves.

### Female egg‐laying behavior

To study whether females have an innate egg‐laying preference for particular host grasses, we used freshly mated females from the F1‐generation in a behavioral assay. The experiment was carried out in the controlled greenhouse conditions (27°C, 65RH) using a randomized block design. Before the experiment, four species of host plants were randomly selected from the stock in such a way that one species from each block (C_3_O, C_3_S, C_4_O, C_4_S) was included in each trial. Specifically, the exact composition of the four plants to be presented simultaneously for a female was randomized within treatment blocks using a random number generator, after which experimental plants for each session were haphazardly picked from the plant stock of different experimental groups. Each plant species was presented five times. Plants were put into a mesh‐covered, circular hanging cage (20 cm in diameter, with a total length of c. 28 cm; Insectopia, Austrey, UK) into which a single randomly chosen gravid female was released at the center. The position of the cage within the growth facility was randomized to control for heterogeneous light, and all plants were healthy and of similar size. The female was allowed to lay eggs for 24 h, after which the number of eggs on each grass species was counted. Fifteen independent trials were run, each with a new female and a new combination of grass species.

### Hatchling establishment success

To examine whether larvae differ in performance on sun or shade grasses with either C_3_ or C_4_ photosynthetic physiology, ten hatchling larvae were put on each of the randomly selected grass plants covered with mesh bags. This was repeated seven times for each of 12 plant species, resulting in 840 larvae entering the experiment. Average instar weights were determined at the start of the experiment, and after 7 days, their weight increase and survival (number of living individuals) was recorded.

To understand which leaf traits were the most important determinants for larval performance, we measured ten representatives of each species and examined two sets of leaf traits. Firstly, in relation to insect feeding accessibility, we measured hairiness, toughness, and waxiness. Hairiness was the number of hairs within a focal area counted using a Zeiss Opton microscope (Oberkochen, Germany) at 25× magnification (a fixed area of *c*. 1 cm^2^). Toughness was measured as the force (in Newtons: 1N = kg × m/s^2^) needed to penetrate the leaf surface using a digital force gauge penetrometer (FH 10; SAUTER GmbH, Balingen, Germany). Waxiness was measured from the abaxial (i.e., under) side of the leaf as a visual assay from 1 to 4 (absent, sparse, moderate, heavy) of leaf wax covering. Secondly, for plant palatability, we measured leaf water content, specific leaf area, and nutrient content. Water content was measured as fresh weight divided by dry weight, and specific leaf area (SLA) as the ratio of leaf area to dry mass. High values of SLA correspond to a large leaf area in relation to dry tissue (and vice versa), therefore robustly characterizing the amount of soft plant tissue that a herbivore can consume per unit time. Leaf area was measured from young leaves using scale‐calibrated digital photographs. Nutrient content was derived from isotope analysis (see below) as the ratio of elemental carbon to nitrogen contents. Leaf traits were further combined using principal component analysis (see below and Table 1).

**Table 1:** Principal component factor loadings of the leaf traits

Subject	Trait	PC1	PC2
Accessibility	Toughness	**0.911**	−0.212
	Waxiness	**0.831**	0.260
	Hairiness	−0.042	**0.918**
Palatability	Water content	−**0.681**	0.077
	Specific leaf area	−**0.890**	−0.358
	C/N – ratio	**0.760**	−0.330

Bold values indicate the component which has higher loading for the trait.

### Habitat preference

Field sites were chosen a priori using satellite and aerial images to detect suitable habitats (Google Earth, Google Inc., Mountain View, CA), after which the areas were visited to confirm the presence of *B. safitza*. The Eastern Cape is a temperate region that receives progressively more rainfall to the east. The region is characterized by open, semiarid grasslands, whereas afromontane forests and coastal thickets provide more humid, shaded habitats. We used three field sites in the Eastern Cape to investigate the occurrence of *B. safitza* across habitats: Bathurst (−33.501522°, 26.773979°), Kapriver (−33.349859°, 26.859918°), and Kasouga (−33.650250°, 26.740267°). The Bathurst site is a riparian bush habitat that is surrounded by land held in common pasturing of animals with more open habitat along its edges. Kapriver is an open grassy hilltop that transitions into riparian forest in a lower lying river gorge. Kasouga is characterized by coastal thickets bordered by pastureland and the sea.

To obtain data on habitat preference, we trapped butterflies in transects running from C_4_‐dominated grasslands to C_3_‐dominated shaded forest understories. At each of three sites, we positioned nine traps: three traps in the open grassland, three at the transition from the grassland to the forest, and three in the fully shaded forest understories. Open grassland was characterized by scattered shrubs and no overstory canopy. The transition from grassland to forest was frequently sharp but occasionally included a transition‐like successional stage. The forest habitat was typically an enclosed dense canopy at a height of approximately 5 m, below which was a shaded understory (*Bicyclus* butterflies tend to keep close to the ground and the understory, as well as near forest edges with scattered‐canopy in tropical Africa, Larsen 2005).

Trapping was conducted once a month for a period of 24 h, during the austral summer from November to April 2015. A day before collecting the butterflies, traps (Megaview, DC0017, Pop‐up Butterfly Bait Trap, cone type) were set up and baited with fermented banana. Occasionally, bait disappeared due to unidentified causes, a potential agency being monkeys. We supplemented trapping effort by catching butterflies with a hand‐net in an area within approximately 10 m of the baited trap at the second visit. Butterflies were stored in envelopes packed into plastic containers for later investigation.

We also studied butterfly dry weights, because weight can be informative in the context of life‐history constraints (Nylin 2013). We predict that butterflies grow to a smaller adult mass if larvae have eaten low‐quality food (such as C_4_, see above), whereas they should grow larger if larvae have eaten better quality food (such as C_3_). Thus, we measured butterfly dry weights by detaching wings from the bodies, drying bodies for 48 h at 40°C, and measuring dry weight using a digital scale (Mettler Toledo MX5, Columbus, OH). Weights were rounded up to the nearest milligram.

### Stable isotope analysis

Stable isotopes of carbon can provide direct evidence of C_3_ and C_4_ host plant use. To measure the relative amount of the heavy stable isotope of carbon (*δ* ^13^C) in our specimens (Cerling et al. 1997), leg tissue was placed into 8 × 5 mm tin capsules, sealed, and loaded into an auto‐sampler. The tissue within the capsule was broken down into its elemental components and analyzed for ^13^C/^12^C using an Elemental Analyser (Costech, Valencia, CA) attached to a mass spectrometer (Thermo DELTA V, Waltman, MA). Samples are continuously purged with helium to prevent contamination with water, oxygen, and nitrogen. The gaseous products produced were separated by a packed gas chromatographic molecular sieve column at a temperature of 90°C and passed into the mass spectrometer via a universal continuous interface (Thermo Conflo IV, Waltman, MA). The mass spectrometer software is programmed to allow the areas under peaks of ^12^CO_2_ and ^13^CO_2_ to be measured, enabling the ^13^C/^12^C isotope ratio to be calculated. Reference standards from IAEA in Vienna were run at intervals throughout the sequence, and these values are used to calibrate to the international standards of ^13^C/^12^C (*δ* ^13^C Vienna‐PDB).

### Statistical analyses

We approach our question in three parts: (1) oviposition experiment, (2) hatchling establishment experiment, and (3) field data. In addition, the summarized values from these independent datasets were used to make generalizations among the separate studies. Analyses were performed in IBM SPSS Statistics (v22), and the R language and environment (version 3.2.1.).

1) We conducted a generalized linear mixed model (GLMM) to test female oviposition preference. The numbers of eggs laid on host plants was used as the dependent variable. As fixed factors, we included the photosynthetic pathway (C_3_, C_4_) and habitat (open, shaded) of the host plants, and their interaction (Table 2). We used a Poisson distribution because the number‐counted data covered a fixed observation period. Female identity and plant species were incorporated as independent random effects in the intercept. GLMM model fitting was carried out with the Laplace approximation, using the lmer function in R PACKAGE LME4 (Bates et al 2015). Model selection was based on the smallest Akaike information criterion (AIC) value both here and in subsequent tests. In GLMMs, the best models are reported according to AIC selection.

**Table 2:** Testing the preference–performance hypothesis of host plants in *Bicyclus safitza*: (A) female oviposition preference, (B) larval growth, and (C) larval survival. In all panels, fixed factors refer to host plant photosynthetic *pathway* (C_3_, C_4_), *habitat* (open, shade), and their interaction (*). The reported models are the best models according to smallest Akaike information criterion (AIC) value. Each test has a random effect incorporated in the intercept: (A) female and plant species and (B–C) plant species. In panels A and C, *test value* refers to *Z*‐statistics, whereas in panel B, it is Student's *t*‐statistic. Significant *P* values are denoted in bold

Source	Estimate	SE	Test value	*P*
(A) Female oviposition				
(Intercept)^a^	0.116	0.294	0.394	0.693
Pathway [C_4_]	−0.958	0.436	−2.198	**0.028**
Habitat [shade]	0.453	0.305	1.484	0.138
Pathway*Habitat	2.568	0.491	5.229	**<0.001**
(B) Larval growth				
(Intercept)^a^	1.202	0.419	2.867	**<0.001**
Pathway [C_4_]	−0.696	0.601	−1.158	0.498
Habitat [shade]	1.584	0.596	2.657	**<0.001**
Pathway*Habitat	2.054	0.858	2.392	**0.010**
(C) Larval survival				
(Intercept)^a^	0.738	0.190	3.870	**<0.001**
Habitat [shade]	0.915	0.248	3.684	**<0.001**

a Intercept includes factor levels: pathway [C_3_] and habitat [open].

2) We also analyzed hatchling establishment success (i.e., larval growth and survival) using GLMM. Growth was measured as the average weight gain of larvae, and survival was the number of larvae alive after 7 days of feeding on the host plant (Table 2). The photosynthetic pathway (C_3_, C_4_) and habitat (open, shaded) of the host plant, and their interaction were included as fixed factors. The plant species was again a random factor. Gaussian distribution for growth (weight gain) and the Poisson distribution for survival (count data) were used. To examine which leaf traits explain hatchling performance on host plants, we conducted principal component analysis (PCA) of host accessibility (leaf toughness, waxiness, hairiness) and palatability (water content, specific leaf area, nutrient content). PCA yielded two significant components, which together explained 75.9% of leaf trait variation. PC1 (55.93%, eigenvalue 3.35) included all the traits apart from hairiness, which in contrast characterized PC2 (19.97%, Eigenvalue 1.19). All other traits had very low weightings in PC2 (see Table 1, factor loadings). We analyzed weight gain, survival, and oviposition preference in grasses with respect to the principal components using linear regression.

To examine whether growth and survival together (2) are associated with the female oviposition preference (1), we conducted PCA of larval performance (i.e., growth and survival), which yielded a single principal component explaining 94.5% of the observed variation and with Eigenvalue 1.19. We then used linear regression to investigate the relationship between oviposition preference and larval performance.

3) We assessed habitat preference as the number of individuals found in different habitats (open grassland, forest fringes, and shaded bush) and tested it against the null expectation of no preference using a chi‐square test. Then, we analyzed carbon isotope signatures extracted from adult butterfly leg tissues, which indicate the larval diet in nature. Specifically, we tested whether adult *δ* ^13^C values show C_3_ or C_4_ characteristic signals against a null expectation of no preference, using a cutoff value of −21 ‰, whereby values lower than −21 ‰ *δ* ^13^C are typical of the utilization of a C_3_ host plant whereas values higher than −21 *δ* ^13^C are typical of a C_4_ diet. In addition, we conducted a linear regression to analyze whether adult *δ* ^13^C values change with habitat or month of capture as predicting factors (Table 3); habitat was included to detect whether host plant use is different between the habitats, and month of capture was included to detect whether seasonality changes the observed host plant preference. Finally, we conducted linear regression on the dry weights of wild‐caught adults to test whether month, habitat, larval diet (predicted from *δ* ^13^C), and sex (Table 3) predict performance in relation to various factors, which may be indicative of resource utilization.

**Table 3:** Linear regressions on stable isotope values of carbon (*δ* ^13^C) and adult dry weight (mg) of *Bicyclus safitza*. Significant *P* values are denoted in bold

Source	Estimate	SE	*t*	*P*
*δ* ^13^C				
Habitat	0.367	0.784	0.469	0.640
Month	−0.281	0.149	−1.882	0.061
Dry weight (mg)				
*δ* ^13^C	−36.473	59.949	−0.608	0.543
Habitat	−172.475	762.635	−0.226	0.821
Month	160.124	146.679	1.092	0.276
Sex	6218.392	636.274	9.773	**<0.001**

## Results

### Female egg‐laying behavior

**Figure 1:**
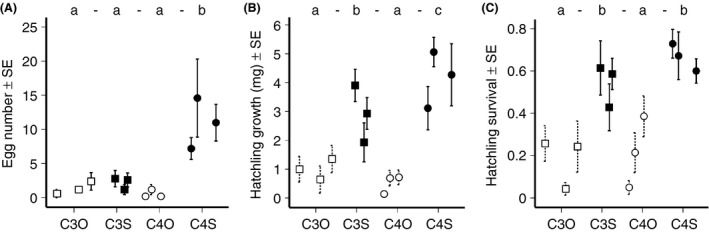
Female oviposition preference links to hatchling establishment of *Bicyclus safitza*. (A) Oviposition preference of females, showing the egg number laid on plants after 24 h. (B) Larval growth on different host plants, showing the average weight gain of larvae in milligrams after 7 days. (C) Larval survival on different host plants after 7 days. In all panels, the minor letters represent significant differences (different letters) between treatment groups (Refer to Table S1 for post hoc comparisons). Open squares: C_3_ plant from open habitat (C_3_O). Solid squares: C_3_ plant from shaded habitat (C_3_S). Open circles: C_4_ plant from open habitat (C_4_O). Solid circles: C_4_ plant from shaded habitat (C_4_S). Groups are each represented by three plant species.

Females showed a clear oviposition preference (Fig. 1A, Table 2). Unexpectedly, they preferred to oviposit on C_4_ shade grasses rather than on either C_4_ grasses from open habitats or C_3_ grasses in general (Table S1). Regressions on plant traits to predict oviposition behavior (Fig. S1) were not significant for either PC1 (leaf traits excluding hairiness) or PC2 (hairiness). We also considered leaf traits separately (Table S2), but additional regression analyses of individual leaf traits to predict female oviposition preference gave similar results (Table S3).

### Hatchling establishment success

**Figure 2:**
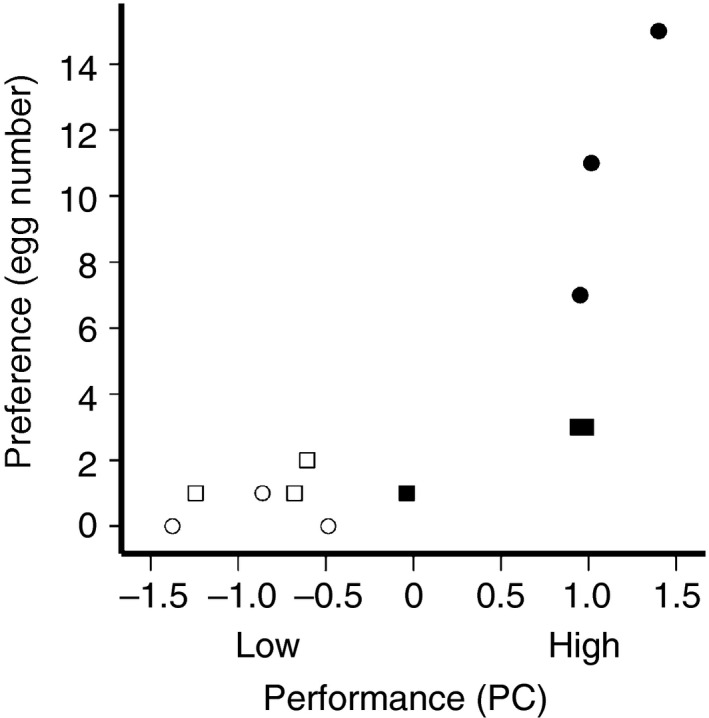
The relationship between female oviposition preference and composite effects of larval growth and survival in *Bicyclus safitza*. Larval performance is shown as principal component (PC); the left‐ and right‐hand ends of the *x*‐axis describe low and high performance, respectively. Treatment groups are as follows. Open squares: C_3_ plant from open habitat (C_3_O). Solid squares: C_3_ plant from shaded habitat (C_3_S). Open circles: C_4_ plant from open habitat (C_4_O). Solid circles: C_4_ plant from shaded habitat (C_4_S). Groups are each represented by three plant species.

Growth rate (mg) was most rapid on C_4_ shade grasses (Fig. 1B), followed by C_3_ shade over C_3_ open habitat grasses; weight gain was least on C_4_ grasses from open habitats (Table S1). Survival was highest on C_4_ shade grasses (Fig. 1C), followed by C_3_ shade grasses over open habitat C_4_ grasses; larvae were least successful on C_3_ grasses from open habitats. In sum, larval growth rate (i.e., weight gain) was significantly higher on C_4_ shade grasses (Fig. 1B, Table 2) than on grasses from any other treatment group, but early larval survival on shade grasses was equally good regardless of the photosynthetic pathway (Fig. 1C, Table 2, Table S1). Nonetheless, overall larval performance (PC for performance, Fig. 2) was associated with female oviposition preference (*F*_1,10_ = 14.59, *P* = 0.003, *R* ^2^ = 0.59); *B* = 3.68, *t* = 3.82, *P* = 0.003).

**Figure 3:**
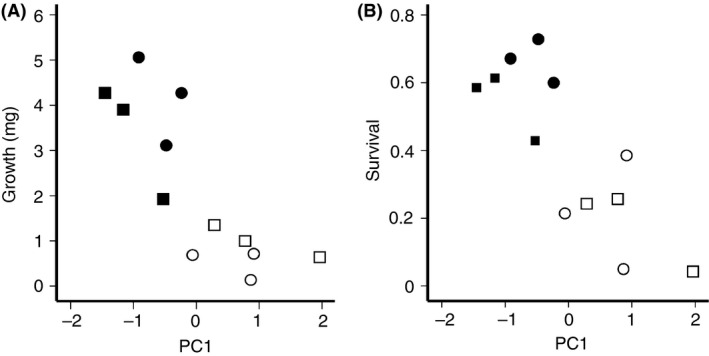
The composite effects of leaf traits (PC1) on hatchling establishment success of *Bicyclus safitza*. See Table 1 for factor loadings. (A) Growth rate of hatchlings measured as mean weight gain (mg) and (B) hatchling survival, both after 7 days on different host plants. Open squares: C_3_ plant from open habitat (C_3_O). Solid squares: C_3_ plant from shaded habitat (C_3_S). Open circles: C_4_ plant from open habitat (C_4_O). Solid circles: C_4_ plant from shaded habitat (C_4_S). Each group is represented by three plant species.

Additionally, we ran multiple regressions to predict larval performance (growth and survival) from the measured leaf traits. Both larval weight (*F*_2,9_ = 13.08, *P* = 0.002, *R* ^2^ = 0.77; *B* = −1.42, *t* = −4.82, *P* = 0.001) and survival (*F*_2,9_ = 9.10, *P* = 0.007, *R* ^2^ = 0.66; *n* = 12, *B* = −0.19, *t* = −4.25, *P* = 0.002) were significantly explained by plant traits (PC1, Fig. 3). Hairiness (PC2) did not have a significant effect either on growth or survival. Weight gain and survival correlated positively with plant specific leaf area (SLA = ratio of leaf area to dry mass), but negatively with waxiness (Fig. 3). The most important leaf trait was toughness, which negatively predicted larval performance (Table S3).

### Habitat preference and isotope signatures

**Figure 4:**
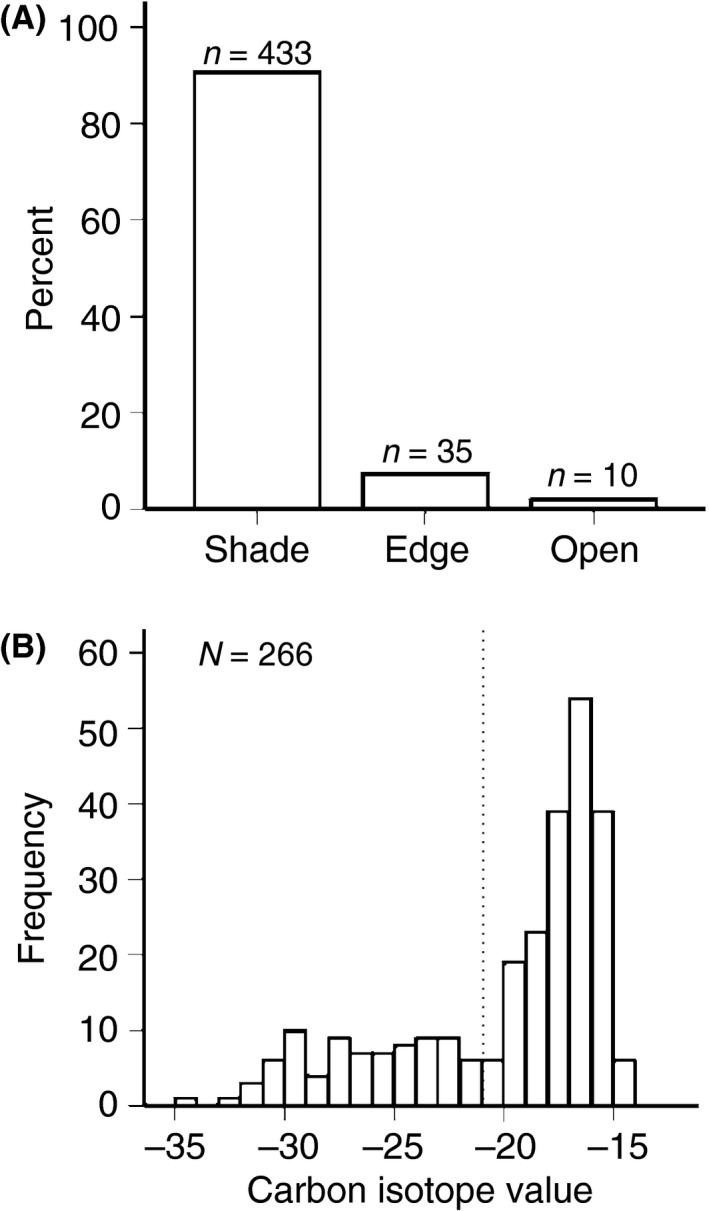
(A) Percentage of *Bicyclus safitza* adults caught from different habitats. (B) Frequency histogram of carbon isotope signatures extracted from adult butterfly leg tissues. The isotope values are indicative to larval diet in the nature. Values lower than −21 *δ* ^13^C (dashed line) are typical to C_3_ (i.e., left‐hand side) whereas values higher than −21 *δ* ^13^C are typical to (i.e., right‐hand side) C_4_ host plant signature.

We found a strong habitat preference (*X* ^2^ = 707.02, *n* = 478, *P* < 0.001) for adults of *B. safitza* (Fig. 4A): 91% were caught in shaded‐understory forest habitats, 7% from the forest edge, and only 2% from open grasslands. We tested whether adult *δ* ^13^C values show C_3_ or C_4_ characteristic signals against the null expectation of no preference and found that butterflies had C_4_ characteristic carbon isotope values significantly more often than expected (*X* ^2^ = 84.48, *n* = 266, *P* < 0.001). Of 266 isotope records, 70% showed a C_4_ characteristic carbon isotope signal, whereas 30% showed a signature indicative of a C_3_ diet (Fig. 4B). Carbon isotope values were unrelated to either the month of capture or the habitat (Table 3). The dry mass of butterflies was also unrelated to these factors. However, males were smaller than females (Table 3).

## Discussion

We show that a preference to lay eggs on C_4_ shade grasses in *B. safitza* is associated with a higher performance of hatchlings. More specifically, the laboratory experiments showed a preference of females to lay eggs on C_4_ shade grasses rather than on either C_4_ grasses from open habitats or C_3_ grasses. The oviposition preference optimized the hatchling growth rate, although hatchling survival on shade grasses was equally good regardless of the photosynthetic pathway. We also show that *B. safitza* butterflies in South Africa usually fly in shade habitats and the majority feed on C_4_ grasses as larvae. We conclude that this pattern may reflect the evolutionary history of *B. safitza*.

*Bicyclus* butterflies are typically found in forests or in close association with forest fringes, but some species occur also in open areas with only few trees (Brakefield and Reitsma 1991; Windig et al. 1994; Roskam and Brakefield 1999; Woodhall 2005). We observed a marked 91 percent preference for shade habitats, which probably reflects the general habitat suitability (Rausher 1979; Hirzel and Le Lay 2008; Suggitt et al. 2011). Although this may be defined by several abiotic (e.g., humidity, temperature, irradiance) and biotic factors (e.g., predators, competition, plant secondary defences), we specifically focused on the host plant ecology. C_3_ grasses often dominate forest understories and the consequent nutritional benefits (Caswell and Reed 1976; Barbehenn et al. 2004a,b) could have limited these butterflies to forest fragments. It is also plausible that adaptations to the abiotic environment (e.g., sun or shade adaptations) may contribute to a host plant's suitability for herbivores. However, according to the selective herbivory hypothesis (Caswell et al. 1973; Caswell and Reed 1976), herbivores should prefer C_3_ plants when they are available. Thus, we predicted that females to prefer C_3_ plants due to their higher palatability over C_4_ plants for their offspring to develop on (Caswell et al. 1973; Heidorn and Joern 1984; Barbehenn et al. 2004a; Christin and Osborne 2014).

Surprisingly, we found that females preferred C_4_ shade grasses. It is considered that female butterflies make oviposition decisions by (1) finding the correct habitat (Friberg et al. 2008), (2) choosing the correct host plant using chemical and/or visual signals, and (3) making the final choice to oviposit using chemical and/or tactile cues (Singer 1971; Thompson and Pellmyr 1991; Singer and Stireman 2001; Nylin et al. 2014; Knolhoff and Heckel 2014; Schäpers et al. 2015). Our preliminary measurements suggest that females may be using short and long wavelengths of reflectance from leaf surfaces to guide the host plant choice. While visual as well as chemical cues seem plausible aids for oviposition choice, it is curious that tactile cues, indicated by leaf trait measurements, did not explain female preference. This may suggest that, in *B. safitza*, the female oviposition preference is linked to selection for larval performance instead, as the performance–preference hypothesis (Jaenike 1978; Valladares and Lawton 1991; West and Cunningham 2002) postulates that maternal oviposition choices should maximize offspring fitness (Leimar and McNamara 2015).

Indeed, hatchlings had their highest weight gain on C_4_ shade grasses. Early larval survival, however, was equally good on shade grasses regardless of the photosynthetic pathway. A potential explanation for the mismatch between female oviposition behavior and larval survival could be that the selective pressures driving the performance–preference are different for females and larvae (Wiklund and Friberg 2009; Nylin et al. 2014; Friberg et al. 2015; Schäpers et al. 2015). Females have to make efficient host plant choices, as searching in the suboptimal habitat is costly in terms of time, energy, and vulnerability (Leimar et al. 2003; Friberg et al. 2008; Wiklund and Friberg 2008). For the larvae, immediate resource suitability is more crucial because of their limited mobility, particularly in early instars, and depends strongly on the maternal oviposition choice (Wiklund and Friberg 2009; Gripenberg et al. 2010; Schäpers et al. 2015). In comparison with open grassland plants, shade grasses have a particularly large specific leaf area and provide softer leaf tissue for herbivores to consume. This is true for both C_3_ and C_4_ shade grasses, although leaf morphology of C_4_ grasses still makes them tougher for herbivores to process than C_3_ leaves. Hairiness seemed to have no effect on larval performance. As shade grasses with either photosynthetic physiology had soft leaves, accessibility seems to be a key for why larvae were surviving equally well on them. Further, adult dry weights did not suggest that a C_4_ (or C_3_) diet led to differential life‐history constraints. However, isotope signatures indicated that C_4_ grasses are disproportionally more frequently consumed than C_3_ grasses, but even so nearly one‐third of individuals sampled from the field showed a C_3_‐characteristic host plant signature.

An intriguing question is how this preference for C_4_ plants evolved, when C_4_ leaves are generally considered to be low‐quality food for herbivores (Caswell et al. 1973; Heckathorn et al. 1999; Barbehenn et al. 2004a). We reason that because abiotic conditions govern plant adaptations to sun or shade environments, a mere comparison between C_3_ or C_4_ plants may be oversimplified. *Bicyclus safitza*, as well as closely related species such as *B. cottrelli* and *B. cooksoni* (Aduse‐Poku et al. 2015), are generally considered to be open habitat species (Larsen 2005), where they mainly utilize C_4_ plants (van Bergen et al. 2016). As C_4_ shade grasses are less tough than C_4_ plants of open grassland, they may have provided a more accessible resource than sun‐adapted grasses, which may have facilitated the preference of *B. safitza* for shade habitats. Plausibly, for a strictly C_3_‐specialist butterfly, consuming C_4_ plants, albeit shade‐adapted species, should be increasingly costly, whereas this would be less so for a butterfly already adapted to consume a C_4_ diet in semi‐open grasslands as this would benefit from a new host plant resource in the shade habitat. A novel use of host plants could thus have provided a mechanism to invade novel ecological niches (cf. Friberg et al. 2008; Hirzel and Le Lay 2008; Nylin et al. 2014), such as is represented for *B. safitza* by the temperate environment at the southernmost tip of its distribution, one which is unlike that of any potential source population further north in tropical Africa. Over time, successful establishment in the new environment is likely to have led to local adaptation and the differentiation of a novel population distinct from the source population (see West‐Eberhard 1989; DeWitt et al. 1998; Pfennig et al. 2010).

In conclusion, plant–herbivore interactions can influence the direction of selection in a grass‐feeding butterfly, *B. safitza*. Based on this work, we postulate future research to test whether these interactions more generally contribute to expansions into new unexploited ecological niches.

## Conflict of Interest

None declared.

## Supporting information


**Figure S1.** The relationship between female oviposition preference and the two principal components of larval performance.

**Table S1.** Between group post hoc comparisons of female oviposition preference, larval growth and survival.

**Table S2.** Average values of leaf traits by treatment group and species.

**Table S3.** Regression analyses of female oviposition preference and larval performance with respect to leaf traits of the host plants.

## References

[ece32235-bib-0001] Aduse‐Poku, K. , O. Brattström , U. Kodandaramaiah , D. C. Lees , P. M. Brakefield , and N. Wahlberg . 2015 Systematics and historical biogeography of the old world butterfly subtribe Mycalesina (Lepidoptera: Nymphalidae: Satyrinae). BMC Evol. Biol. 15:167. 26289424 10.1186/s12862-015-0449-3 PMC4545879

[ece32235-bib-0002] Barbehenn, R. V. , and E. A. Bernays . 1992 Relative nutritional quality of C3 and C4 grasses for a graminivorous lepidopteran, Paratrytone melane (Hesperiidae). Oecologia 92:97–103. 10.1007/BF00317268 28311818

[ece32235-bib-0003] Barbehenn, R. V. , Z. Chen , D. N. Karowe , and A. Spickard . 2004a C3 grasses have higher nutritional quality than C4 grasses under ambient and elevated atmospheric CO_2_ . Glob. Change Biol. 10:1565–1575.

[ece32235-bib-0004] Barbehenn, R. V. , D. N. Karowe , and A. Spickard . 2004b Effects of elevated atmospheric CO_2_ on the nutritional ecology of C3 and C4 grass‐feeding caterpillars. Oecologia 140:86–95. 15118901 10.1007/s00442-004-1572-9

[ece32235-bib-0005] Beerling, D. J. , and C. P. Osborne . 2006 The origin of the savanna biome. Glob. Change Biol. 12:2023–2031.

[ece32235-bib-0006] van Bergen, E. , H. S. Barlow , O. Brattström , H. Griffiths , U. Kodandaramaiah , C. P. Osborne , et al. 2016 The stable isotope ecology of mycalesine butterflies: implications for plant‐insect co‐evolution. Funct. Ecol. DOI: 10.1111/1365‐2435.12673

[ece32235-bib-0007] Boutton, T. W. , G. N. Cameron , and B. N. Smith . 1978 Insect herbivory on C3 and C4 grasses. Oecologia 32:21–32. 10.1007/BF00344568 28309224

[ece32235-bib-0008] Brakefield, P. M. , and N. Reitsma . 1991 Phenotypic plasticity, seasonal climate and the population biology of Bicyclus butterflies (Satyridae) in Malawi. Ecol. Entomol. 16:291–303.

[ece32235-bib-0059] Douglas, Bates , Martin Maechler , Ben Bolker , Steve Walker . 2015 Fitting Linear Mixed‐Effects Models Using lme4. Journal of Statistical Software, 67(1):1–48.

[ece32235-bib-0009] Caswell, H. , and F. C. Reed . 1976 Plant‐herbivore interactions – The indigestibility of C4 bundle sheat cells by grasshoppers. Oecologia 26:151–156. 10.1007/BF00582893 28309259

[ece32235-bib-0010] Caswell, H. , F. C. Reed , S. N. Stephenson , and P. Werner . 1973 Photosynthetic pathways and selective herbivory: a hypothesis. Am. Nat., 107:465–480.

[ece32235-bib-0011] Cerling, T. E. , J. M. Harris , B. J. Macfadden , M. G. Leakey , J. Quadek , V. Eisenmann , et al. 1997 Global vegetation change through the Miocene/Pliocene boundary. Nature 389:153–158.

[ece32235-bib-0012] Christin, P.‐A. , and C. P. Osborne . 2014 The evolutionary ecology of C4 plants. New Phytol. 204:765–781. 25263843 10.1111/nph.13033

[ece32235-bib-0013] DeWitt, T. J. , A. Sih , and D. S. Wilson . 1998 Costs and limits of phenotypic plasticity. Trends Ecol. Evol. 13:77–81. 21238209 10.1016/s0169-5347(97)01274-3

[ece32235-bib-0014] Edwards, E. J. , C. P. Osborne , C. A. E. Strömberg , S. A. Smith , W. J. Bond , P.‐A. Christin , et al. 2010 The origins of C4 grasslands: integrating evolutionary and ecosystem science. Science 328:587–591. 20431008 10.1126/science.1177216

[ece32235-bib-0015] Ehleringer, J. R. , and R. K. Monson . 1993 Evolutionary and ecological aspects of photosynthetic pathway variation. Annu. Rev. Ecol. Syst. 24:411–439.

[ece32235-bib-0016] Fagerstone, K. A. , and O. Williams . 1982 Use of C3 and C4 plants by black‐tailed prairie dog. J. Mammal. 63:328–331.

[ece32235-bib-0017] Friberg, M. , M. Olofsson , D. Berger , B. Karlsson , and C. Wiklund . 2008 Habitat choice precedes host plant choice – Niche separation in a species pair of a generalist and a specialist butterfly. Oikos 117:1337–1344.

[ece32235-bib-0018] Friberg, M. , D. Posledovich , and C. Wiklund . 2015 Decoupling of female host plant preference and offspring performance in relative specialist and generalist butterflies. Oecologia, 118:1–1192. 10.1007/s00442-015-3286-6 25783488

[ece32235-bib-0019] Gripenberg, S. , P. J. Mayhew , M. Parnell , and T. Roslin . 2010 A meta‐analysis of preference‐performance relationships in phytophagous insects. Ecol. Lett. 13:383–393. 20100245 10.1111/j.1461-0248.2009.01433.x

[ece32235-bib-0020] Heckathorn, S. A. , S. J. McNaughton , and J. S. Coleman . 1999 C4 plants and herbivory Pp. 285‐312 *in* Sage R. F. and Monson R. K., eds. C4 plant biology. Academic Press, San Diego, CA, USA.

[ece32235-bib-0021] Heidorn, T. , and A. Joern . 1984 Differential herbivory on C3 versus C4 grasses by the grasshopper Ageneotettix deorum (Orthoptera: Acrididae). Oecologia 65:19–25. 10.1007/BF00384457 28312104

[ece32235-bib-0022] Hirzel, A. H. , and G. Le Lay . 2008 Habitat suitability modelling and niche theory. J. Appl. Ecol. 45:1372–1381.

[ece32235-bib-0023] Jaenike, J. 1978 On optimal oviposition behavior in phytophagous insects. Theor. Popul. Biol. 14:350–356. 751265 10.1016/0040-5809(78)90012-6

[ece32235-bib-0024] Knolhoff, L. M. , and D. G. Heckel . 2014 Behavioral assays for studies of host plant choice and adaptation in herbivorous insects. Annu. Rev. Entomol. 59:263–278. 24160429 10.1146/annurev-ento-011613-161945

[ece32235-bib-0025] Laetsch, W. M. 1974 The C4 syndrome: a structural analysis. Annu. Rev. Plant Physiol. 25:27–52.

[ece32235-bib-0026] Larsen, T. B. 2005 Butterflies of West Africa. Stenstrup, Denmark.

[ece32235-bib-0027] Leimar, O. , and J. M. McNamara . 2015 The evolution of transgenerational integration of information in heterogeneous environments. Am. Nat. 185:E55–E69. 25674697 10.1086/679575

[ece32235-bib-0028] Leimar, O. , U. Norberg , and C. Wiklund . 2003 Habitat preference and habitat exploration in two species of satyrine butterflies. Ecography 26:474–480.

[ece32235-bib-0029] Massey, F. P. , and S. E. Hartley . 2006 Experimental demonstration of the antiherbivore effects of silica in grasses: impacts on foliage digestibility and vole growth rates. Proc. R. Soc. B Biol. Sci. 273:2299–2304. 10.1098/rspb.2006.3586 PMC1636089 16928631

[ece32235-bib-0030] Massey, F. P. , R. A. Ennos , and S. E. Hartley . 2007 Grasses and the resource availability hypothesis: the importance of silica‐based defences. J. Ecol. 95:414–424.

[ece32235-bib-0031] Nylin, S. 2013 Induction of diapause and seasonal morphs in butterflies and other insects: knowns, unknowns and the challenge of integration. Physiol. Entomol. 38:96–104. 23894219 10.1111/phen.12014 PMC3712473

[ece32235-bib-0032] Nylin, S. , J. Slove , and N. Janz . 2014 Host plant utilization, host range oscillations and diversification in nymphalid butterflies: a phylogenetic investigation. Evolution 68:105–124. 24372598 10.1111/evo.12227 PMC3912913

[ece32235-bib-0033] Osborne, C. P. , and D. J. Beerling . 2006 Nature's green revolution: the remarkable evolutionary rise of C4 plants. Philos. Trans. R. Soc. Lond. B Biol. Sci. 361:173–194. 16553316 10.1098/rstb.2005.1737 PMC1626541

[ece32235-bib-0034] Peña, C. , and N. Wahlberg . 2008 Prehistorical climate change increased diversification of a group of butterflies. Biol. Lett. 4:274–278. 18364308 10.1098/rsbl.2008.0062 PMC2610051

[ece32235-bib-0035] Pfennig, D. W. , M. A. Wund , E. C. Snell‐Rood , T. Cruickshank , C. D. Schlichting , and A. P. Moczek . 2010 Phenotypic plasticity's impacts on diversification and speciation. Trends Ecol. Evol. 25:459–467. 20557976 10.1016/j.tree.2010.05.006

[ece32235-bib-0036] Rausher, M. D. 1979 Larval habitat suitability and oviposition preference in three related butterflies. Ecology 60:503–511.

[ece32235-bib-0037] Ripley, B. S. , M. E. Gilbert , D. G. Ibrahim , and C. P. Osborne . 2007 Drought constraints on C4 photosynthesis: stomatal and metabolic limitations in C3 and C4 subspecies of Alloteropsis semialata. J. Exp. Bot. 58:1351–1363. 17322550 10.1093/jxb/erl302

[ece32235-bib-0038] Roskam, J. C. , and P. M. Brakefield . 1999 Seasonal polyphenism in Bicyclus (Lepidoptera: Satyridae) butterflies: different climates need different cues. Biol. J. Linn. Soc. 66:345–356.

[ece32235-bib-0039] Sage, R. F. 2004 The evolution of C4 photosynthesis. New Phytol. 161:341–370. 10.1111/j.1469-8137.2004.00974.x 33873498

[ece32235-bib-0040] Schäpers, A. , S. Nylin , M. A. Carlsson , and N. Janz . 2015 Specialist and generalist oviposition strategies in butterflies: maternal care or precocious young? Oecologia 180:335–343. 26141793 10.1007/s00442-015-3376-5

[ece32235-bib-0041] Singer, M. 1971 Evolution of food‐plant preference in the butterfly Euphydryas editha. Evolution 25:383–389. 10.1111/j.1558-5646.1971.tb01892.x 28563107

[ece32235-bib-0042] Singer, M. , and J. Stireman . 2001 How foraging tactics determine host‐plant use by a polyphagous caterpillar. Oecologia 129:98–105. 10.1007/s004420100707 28547072

[ece32235-bib-0043] Spriggs, E. L. , P. A. Christin , and E. J. Edwards . 2014 C4 photosynthesis promoted species diversification during the miocene grassland expansion. PLoS ONE, 9:e97722. 24835188 10.1371/journal.pone.0097722 PMC4023962

[ece32235-bib-0044] Suggitt, A. J. , P. K. Gillingham , J. K. Hill , B. Huntley , W. E. Kunin , D. B. Roy , et al. 2011 Habitat microclimates drive fine‐scale variation in extreme temperatures. Oikos 120:1–8.

[ece32235-bib-0045] Taylor, S. H. , S. P. Hulme , M. Rees , B. S. Ripley , F. I. Woodward , and C. P. Osborne . 2010 Ecophysiological traits in C3 and C4 grasses: a phylogenetically controlled screening experiment. New Phytol. 185:780–791. 20002318 10.1111/j.1469-8137.2009.03102.x

[ece32235-bib-0046] Taylor, S. H. , B. S. Ripley , T. Martin , L.‐A. De‐Wet , F. I. Woodward , and C. P. Osborne . 2014 Physiological advantages of C4 grasses in the field: a comparative experiment demonstrating the importance of drought. Glob. Change Biol. 20:1992–2003. 10.1111/gcb.12498 PMC4237462 24677339

[ece32235-bib-0047] Thompson, J. N. 1988 Evolutionary ecology of the relationship between oviposition preference and performance of off spring in phytophagons insects. Entomol. Exp. Appl. 47:3–14.

[ece32235-bib-0048] Thompson, J. N. , and O. Pellmyr . 1991 Evolution of oviposition behavior and host preference in Lepidoptera. Annu. Rev. Entomol. 36:65–89.

[ece32235-bib-0049] Valladares, G. , and J. H. Lawton . 1991 Host‐plant selection in the holly leaf‐miner: does mother know best? J. Anim. Ecol. 60:227–240.

[ece32235-bib-0050] West, S. A. , and J. P. Cunningham . 2002 A general model for host plant selection in phytophagous insects. J. Theor. Biol., 214:499–513. 11846605 10.1006/jtbi.2001.2475

[ece32235-bib-0051] West‐Eberhard, M. J. 1989 Phenotypic plasticity and the origins of diversity. Annu. Rev. Ecol. Syst. 20:249–278.

[ece32235-bib-0052] Wiklund, C. , and M. Friberg . 2008 Enemy‐free space and habitat‐specific host specialization in a butterfly. Oecologia 157:287–294. 18607636 10.1007/s00442-008-1077-z

[ece32235-bib-0053] Wiklund, C. , and M. Friberg . 2009 The evolutionary ecology of generalization: among‐year variation in host plant use and offspring survival in a butterfly. Ecology 90:3406–3417. 20120809 10.1890/08-1138.1

[ece32235-bib-0054] Windig, J. J. , P. M. Brakefield , N. Reitsma , and J. G. M. Wilson . 1994 Seasonal polyphenism in the wild: survey of wing patterns in five species of Bicyclus butterflies in Malawi. Ecol. Entomol. 19:285–298.

[ece32235-bib-0055] Woodhall, S. 2005 Field guide to butterflies of South Africa. Struik Publishers, Cape Town, South Africa.

