# Supplementary figures and images for: Preference for C4 shade grasses increases hatchling performance in the butterfly, Bicyclus safitza
Source: Ecol Evol. 2016 Jun 29;6(15):5246–55. doi: 10.1002/ece3.2235 (PMC4984501; doi:10.1002/ece3.2235)

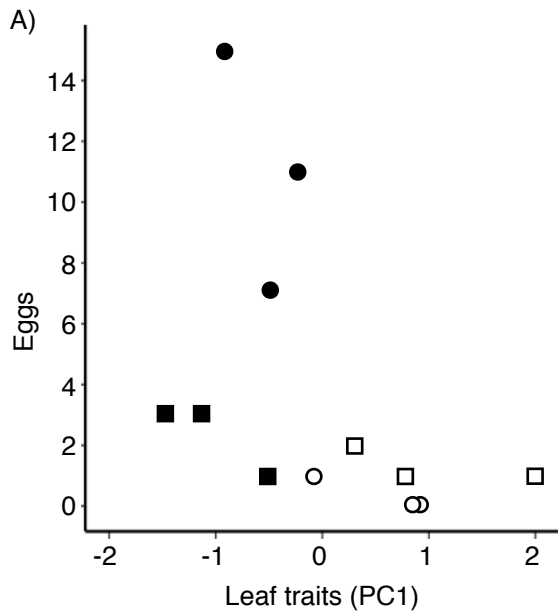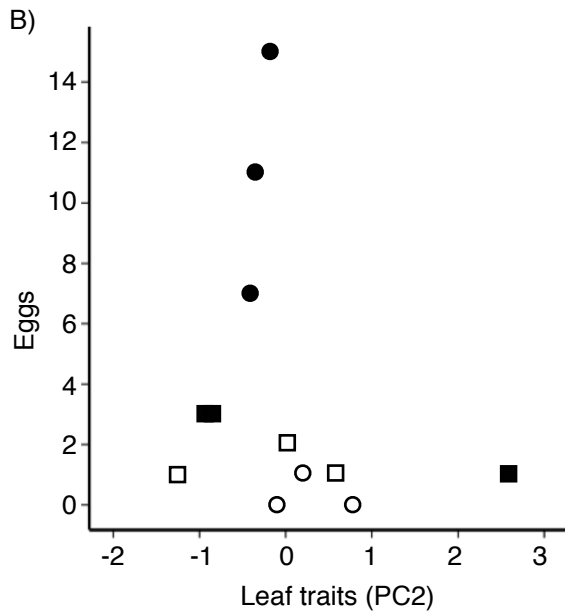

Supplement: Supplementary file 1 — Figure S1. The relationship between female oviposition preference and the two principal components of larval performance. [file ECE3-6-5246-s001.pdf]
