# Supplementary material for: Preference for C4 shade grasses increases hatchling performance in the butterfly, Bicyclus safitza
Source: Ecol Evol. 2016 Jun 29;6(15):5246–55. doi: 10.1002/ece3.2235 (PMC4984501; doi:10.1002/ece3.2235)
Supplement: Supplementary file 2 — Table S1. Between group post hoc comparisons of female oviposition preference, larval growth and survival. [file ECE3-6-5246-s002.docx]

**Table S1** Between group post hoc comparisons of female oviposition preference, larval growth and survival. ANOVA based Tukey’s multiple comparisons of means (diff) with 95% confidence levels (LWR, UPR) are reported. Significant p-values are bolded.

| Source | Diff | LWR | UPR | p |
| --- | --- | --- | --- | --- |
| Oviposition preference |  |  |  |  |
| C4O – C3O | -0.86 | -5.19 | 3.45 | 0.951 |
| C3S – C3O | 0.80 | -3.52 | 5.12 | 0.961 |
| C4S – C3O | 9.53 | 5.20 | 13.85 | **<0.001** |
| C3S – C4O | 1.66 | -2.65 | 5.99 | 0.738 |
| C4S – C4O | 10.40 | 6.07 | 14.72 | **<0.001** |
| C4S – C3S | 8.73 | 4.40 | 13.05 | **<0.001** |
| Larval growth |  |  |  |  |
| C4O – C3O | -0.62 | -1.81 | 0.57 | 0.530 |
| C3S – C3O | 1.76 | 0.53 | 2.99 | **0.001** |
| C4S – C3O | 3.02 | 1.79 | 4.25 | **<0.001** |
| C3S – C4O | 2.38 | 1.15 | 3.61 | **<0.001** |
| C4S – C4O | 3.64 | 2.41 | 4.87 | **<0.001** |
| C4S – C3S | 1.26 | 0.01 | 2.51 | **0.049** |
| Larval survival |  |  |  |  |
| C4O – C3O | 0.04 | -1.94 | 2.03 | 0.999 |
| C3S – C3O | 3.30 | 1.26 | 5.34 | **<0.001** |
| C4S – C3O | 4.65 | 2.61 | 6.69 | **<0.001** |
| C3S – C4O | 3.25 | 1.21 | 5.30 | **<0.001** |
| C4S – C4O | 4.60 | 2.56 | 6.65 | **<0.001** |
| C4S – C3S | 1.35 | -0.73 | 3.43 | 0.333 |
