# Supplementary material for: Preference for C4 shade grasses increases hatchling performance in the butterfly, Bicyclus safitza
Source: Ecol Evol. 2016 Jun 29;6(15):5246–55. doi: 10.1002/ece3.2235 (PMC4984501; doi:10.1002/ece3.2235)
Supplement: Supplementary file 3 — Table S2. Average values of leaf traits by treatment group and species. [file ECE3-6-5246-s003.docx]

**Table S2** Average values of leaf traits by treatment group and species. The plant species in treatment groups are as follows. C_3_O: *Allopteropsis semialata ssp. eckloniana, Panicum ecklonii, Merxmuellera disticha*. C_3_S: *Ehrharta erecta, Oplismenus hirtellus, Panicum aequinerve.* C_4_O: *Allopteropsis semialata ssp. semialata, Hyparrhenia hirta, Brahiaria serrata*. C_4_S: *Brachiaria chusquoides, Dactyloctenium australe, Panicum deustum*. Ten representatives of each species were used. Toughness is measured as the force needed to penetrate the leaf surface (1N = kg * m/s^2^). Hairiness is the number of hairs within a fixed area of abaxial leaf surface (hairs / 1 cm^2^). Waxiness is measured as a visual assay from 1 to 4 (absent, sparse, moderate, heavy) of leaf wax covering. Water content is a ratio of fresh and dry weight, measured as fresh weight divided by dry weight (in milligrams). Specific leaf area (SLA) was measured as the ratio of leaf area to dry mass (cm^2^ / mg). Nutrient content was derived from isotope analysis as the ratio of elemental carbon to nitrogen contents (C/N). For full details refer to methods.

| Group | Species | Toughness | Waxiness | Hairiness | Water content | Specific leaf area | C / N - ratio |
| --- | --- | --- | --- | --- | --- | --- | --- |
| C_3_O | *A. sem* C_3_ | 3.40 | 3 | 39 | 2.74 | 295.93 | 31.79 |
|  | *P .eck* | 3.25 | 3 | 1 | 3.56 | 566.20 | 15.02 |
|  | *M. dis* | 6.75 | 3 | 2 | 1.29 | 185.02 | 55.72 |
| C_3_S | *E. ere* | 1.04 | 1 | 1 | 3.57 | 3248.59 | 14.66 |
|  | *O. hir* | 1.25 | 2 | 108 | 4.02 | 1361.10 | 12.11 |
|  | *P. aeq* | 1.28 | 1 | 12 | 3.53 | 4803.65 | 12.55 |
| C_4_O | *A. sem* C_4_ | 5.71 | 4 | 2 | 3.46 | 762.02 | 16.95 |
|  | *H. hir* | 3.28 | 3 | 35 | 0.33 | 80.10 | 18.16 |
|  | *B. ser* | 3.25 | 2 | 26 | 3.51 | 1577.08 | 17.88 |
| C_4_S | *B. chu* | 1.38 | 2 | 1 | 3.15 | 2521.88 | 15.90 |
|  | *D. aus* | 1.35 | 2 | 5 | 5.72 | 2582.49 | 14.25 |
|  | *P. deu* | 1.88 | 2 | 4 | 3.08 | 1910.54 | 18.43 |
