# Supplementary material for: Preference for C4 shade grasses increases hatchling performance in the butterfly, Bicyclus safitza
Source: Ecol Evol. 2016 Jun 29;6(15):5246–55. doi: 10.1002/ece3.2235 (PMC4984501; doi:10.1002/ece3.2235)
Supplement: Supplementary file 4 — Table S3. Regression analyses of female oviposition preference and larval performance with respect to leaf traits of the host plants. [file ECE3-6-5246-s004.docx]

**Table S3** Regression analyses of female oviposition preference and larval performance with respect to leaf traits of the host plants. When the leaf traits are analysed as a group of separate values for each trait, rather than using principal components (refer to methods), the traits do not significantly predict the number of eggs that female oviposit (F_6,5_ = 3.19, p = 0.112, R^2^ = 0.79), albeit a significant coefficient for toughness occurs. In contrast, both larval growth (F_6,5_ = 6.80, p = 0.026, R^2^ = 0.89) and survival (F_6,5_ = 20.57, p = 0.002, R^2^ = 0.96) were significantly predicted by host plant leaf traits. The coefficients for the leaf traits are reported below and significant p-values are denoted in bold.

| Leaf trait | B | SE | t | p |
| --- | --- | --- | --- | --- |
| Oviposition preference |  |  |  |  |
| Hairiness | -0.72 | 0.03 | -1.86 | 0.121 |
| Toughness | -3.67 | 1.24 | -2.96 | **0.031** |
| Waxiness | -4.26 | 2.51 | 1.69 | 0.151 |
| Water content | 1.16 | 1.01 | 1.15 | 0.301 |
| Specific leaf area | <0.01 | <0.01 | 0.74 | 0.489 |
| C/N - ratio | 0.29 | 0.13 | 2.09 | 0.090 |
| Larval growth |  |  |  |  |
| Hairiness | -0.01 | 0.01 | -1.53 | 0.184 |
| Toughness | -0.95 | 0.33 | -2.88 | **0.034** |
| Waxiness | 0.50 | 0.67 | 0.75 | 0.482 |
| Water content | 0.19 | 0.26 | 0.70 | 0.511 |
| Specific leaf area | <0.01 | <0.01 | 1.13 | 0.309 |
| C/N - ratio | 0.06 | 0.03 | 1.86 | 0.121 |
| Larval survival |  |  |  |  |
| Hairiness | <-0.01 | <0.01 | -2.00 | 0.101 |
| Toughness | -0.18 | 0.02 | -6.79 | **0.001** |
| Waxiness | 0.13 | 0.05 | 2.37 | 0.063 |
| Water content | -0.03 | 0.02 | -1.79 | 0.132 |
| Specific leaf area | <0.01 | <0.01 | 2.04 | 0.096 |
| C/N - ratio | <0.01 | <0.01 | 2.38 | 0.063 |
